# Percutaneous Left Atrial Appendage Closure in Patients with Non-Valvular Atrial Fibrillation and End-Stage Renal Disease on Hemodialysis: A Case Series

**DOI:** 10.3390/medicina60020231

**Published:** 2024-01-29

**Authors:** Elena Basabe, José C. De La Flor, Virginia López de la Manzanara, Luis Nombela-Franco, Carlos Narváez-Mejía, Leónidas Cruzado, Daniel Villa, Rocío Zamora, Manuel Tapia, Miguel Ángel Sastre, Edurne López Soberón, José A. Herrero Calvo, Alfonso Suárez, David Martí Sánchez

**Affiliations:** 1 Department of Cardiology, Hospital Central Defense Gómez Ulla, 28047 Madrid, Spain; mtapma1@mde.es (M.T.); msaspe1@mde.es (M.Á.S.); elopsob@fn.mde.es (E.L.S.); csuacue@oc.med.es (A.S.); dmars16@oc.mde.es (D.M.S.); 2 Department of Nephrology, Hospital Central Defense Gómez Ulla, 28047 Madrid, Spain; jflomer@mde.es; 3 Department of Nephrology, Hospital Clínico San Carlos, 28040 Madrid, Spain; virginia.lopezmanzanara@salud.madrid.org (V.L.d.l.M.); joseantonio.herrero@salud.madrid.org (J.A.H.C.); 4 Department of Cardiology, Hospital Clínico San Carlos, 28040 Madrid, Spain; luis.nombela@salud.madrid.org; 5 Department of Nephrology, Hospital Universitario Puerta del Mar, 11009 Cádiz, Spain; ceduardo.narvaez.sspa@juntadeandalucia.es; 6 Department of Nephrology, Hospital General Elche, 03203 Elche, Spain; cruzado-leo@gva.es; 7 Department of Nephrology, Clínica Universidad de Navarra, 31008 Navarra, Spain; devillah@unav.es; 8 Department of Nephrology, Hospital Universitario General Villalba, 28400 Madrid, Spain; rocio.zamora@hgvillalba.es

**Keywords:** atrial fibrillation, end-stage renal disease, hemodialysis, percutaneous left atrial appendage closure

## Abstract

Non-valvular atrial fibrillation (NVAF) is the most common cardiac arrhythmia in the general population, and its prevalence increases among patients with chronic kidney disease (CKD) undergoing hemodialysis. This population presents high risk of both hemorrhagic and thrombotic events, with little evidence regarding the use of oral anticoagulation treatment (OAT) and multiple complications arising from it; however, stroke prevention with percutaneous left atrial appendage closure (LAAC) is an alternative to be considered. We retrospectively describe the safety and efficacy of percutaneous LAAC in eight patients with NVAF and CKD on hemodialysis during a 12-month follow-up. The mean age was 78.8 years (range 64–86; SD ± 6.7), and seven patients were male. The mean CHA_2_DS_2_-VAS_C_ and HAS-BLED scores were high, 4.8 (SD ± 1.5) and 3.8 (SD ± 1.3), respectively. Seventy-five percent of the patients were referred for this intervention due to a history of major bleeding, with gastrointestinal bleeding being the most common type, while the remaining twenty-five percent of the patients were referred because of a high risk of bleeding. The percutaneous LAAC procedure was successfully completed in 100% of the patients, with complete exclusion of the appendage without complications or leaks exceeding 5 mm. There was one death not related to the procedure four days after the intervention. Among the other seven patients, no deaths, cardioembolic events or major bleeding were reported during the follow-up period. In our sample, percutaneous LAAC appears to be a safe and effective alternative to anticoagulation in patients with NVAF and CKD on hemodialysis.

## 1. Introduction

Non-valvular atrial fibrillation (NVAF) is the most common cardiac arrhythmia in the general population, and its prevalence increases among patients with chronic kidney disease (CKD) [1]. In patients with end-stage renal disease (ESRD) receiving maintenance hemodialysis (MHD), the prevalence of this association is notably higher, with figures ranging between 13% and 27% [2,3,4]. The incidence of atrial fibrillation (AF) is observed to be higher in patients receiving MHD compared to those undergoing peritoneal dialysis (PD) [5,6].

Remarkably, almost half of patients with atrial fibrillation (AF) will develop some degree of CKD. Furthermore, the presence of CKD is intrinsically linked to an elevated risk of stroke, thromboembolism and major bleeding [7]. CKD is an independent risk factor for AF, which increases as the glomerular filtration rate (GFR) decreases and/or proteinuria increases [5]. In addition, the electrolytic disorders that present during hemodialysis (HD) sessions in patients with ESRD on renal replacement therapy (RRT) also increase this risk [8]. There are multiple comorbidities associated with CKD that also increase the risk of AF, such as altered cardiac structure, endothelial dysfunction, vascular calcification, premature atherosclerosis or increased activity of the renin angiotensin and adrenergic systems. Therefore, it is alarming that patients with CKD and AF have a high mortality risk [6]. Several mechanisms establish a connection between AF and CKD. One notable mechanism involves the presence of elevated levels of inflammatory markers in the early stages of CKD, and as CKD progresses, this inflammation becomes more pronounced [9]. This inflammation has been identified as a significant contributor to the development and persistence of AF [10]. On the other hand, the activation of the renin–angiotensin–aldosterone system (RAAS) serves as another crucial link between AF and CKD. The heightened production of reactive oxygen species, the upregulation of cytokines, cell adhesion molecules and profibrotic growth factors and the induction of extracellular matrix proteins among others are some of the processes through which the RAAS plays a role in the pathogenesis and progression of CKD [11,12,13]. Additionally, the RAAS may contribute to the development of AF by promoting atrial pressure overload and fibrosis [14].

Patients with AF and CKD, have a significantly higher risk of thromboembolism and bleeding at the same time [6]. Most AF patients on MHD have a high CHA_2_DS_2_-VAS_c_ score and, simultaneously, a high HAS-BLED score [6]; thus, its management is challenging for clinicians. Recently, an RCT showed lack of superiority of DOACS over VKA in MHD, with both treatments resulting in unacceptable bleeding rates above 25% per year [15]. Thus, as stated in the guidelines of the European Society of Cardiology, oral anticoagulation treatment (OAT) is not recommended in patients with GFR < 15 mL/min due to the scarce evidence regarding its safety and efficacy [16]. LAAC has emerged as an attractive therapy for challenging cases, and the patient with ESRD is conceptually a clear potential beneficiary, as stated in the consensus documents [17]. However, while there are multiple studies that support the efficacy and safety of this technique in the general population, there is little evidence on its results among patients undergoing MHD. 

Therefore, this study aimed to address this significant knowledge gap by investigating the safety and efficacy of LAAC in this particularly high-risk group of patients. In light of the heightened risks and limited therapeutic alternatives, understanding the potential benefits and risks associated with LAAC in the context of CKD and AF is of paramount importance, both for the improvement of clinical outcomes and the optimization of patient care in this complex, at-risk population.

## 2. Materials and Methods

Our study was carried out in the hemodialysis and interventional cardiology units at two academic centers (Hospital Central de la Defensa Gómez Ulla and Hospital Clínico San Carlos in Madrid, Spain). Information was retrospectively collected from both new and NVAF consecutive patients with ESRD on MHD who had a relative contraindication for using oral anticoagulants. These patients met the criteria for percutaneous left atrial appendage closure (LAAC) according to the 2014 EHRA/EAPCI expert consensus statement on catheter-based left atrial appendage occlusion [18]. The study period spanned from 2020 to 2023. 

The LAAC procedure was performed by experienced interventional cardiologists and under general anesthesia, with guidance from fluoroscopy and transesophageal echocardiography. The size of the LAAC devices (Watchman FLX) was based on the specific anatomical features of each patient and the experience of the interventional cardiologist.

We conducted a descriptive analysis of the baseline characteristics and outcomes of the patient cohort. Continuous variables are reported as the mean, range and standard deviation (SD), and categorical variables are presented as frequencies and percentages.

The primary outcome measure was the success of the LAAC intervention, defined as successful implantation of the device in the left atrial appendage (LAA), with no significant leak (>5 mm) and absence of immediate complications. Reportable adverse events included death, myocardial infarction, ischemic or hemorrhagic stroke, transient ischemic attack, systemic embolism, air embolization, device displacement or embolization, significant pericardial effusion and major bleeding. Long-term patient evolution was evaluated over a follow-up period of 12 months.

## 3. Results

Baseline characteristics: Table 1 provides an overview of the baseline characteristics of the study cohort. Eight patients with ESRD on hemodialysis who underwent a percutaneous LAAC were included in the study. The mean age was 78.8 years (range 64–86; SD ± 6.7), and most of the patients were male (*n* = 7). Two patients had permanent NVAF, whereas in the other six, it was persistent or paroxysmal. The mean CHA_2_DS_2_-VASc score was 4.8 (SD ± 1.5), indicating a high risk of thromboembolic events. The mean HAS-BLED score was 3.8 (SD ± 1.3), reflecting a high risk of bleeding. With the exception of one patient, all presented major cardiovascular risk factors such as hypertension or diabetes mellitus. The patients were referred for the intervention by the treating nephrologist in the hemodialysis unit; seventy-five percent of the patients were referred due to a history of major bleeding, with gastrointestinal bleeding being the most common type, while the remaining twenty-five percent of the patients were referred because of a high risk of bleeding. One patient had a previous ischemic stroke before LAAC. Six patients were on anticoagulation therapy, with half on VKA and the other half on DOACs. The remaining two received double antiplatelet therapy (DAPT) secondary to an acute coronary syndrome during the previous twelve months. The average left ventricular ejection fraction (LVEF) was 50.4 ± 11.5%, and two patients had moderate to severe left ventricular dysfunction.

**Figure 1:**
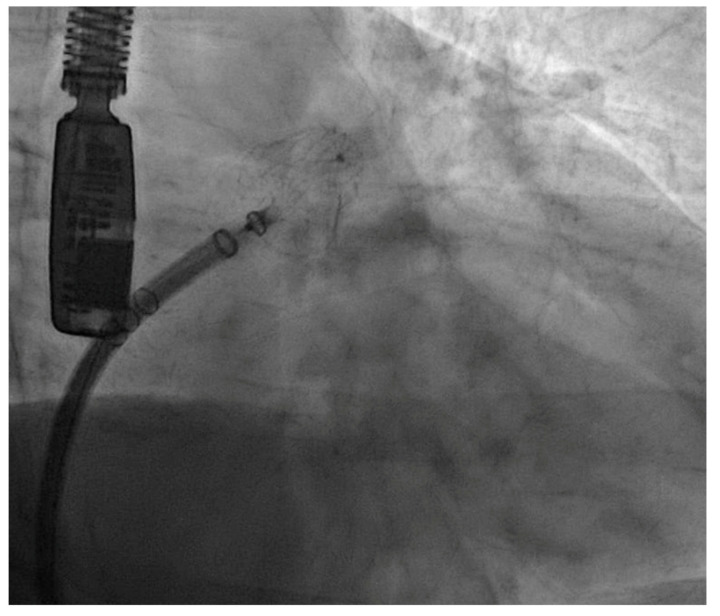
Angiographic image of a Watchman FLX device successfully implanted in one of our patients.

Intervention success: As shown in Table 2, the percutaneous LAAC procedure was successfully completed in 100% of the patients, with complete exclusion of the appendage without complications or leaks exceeding 5 mm. A Watchman FLX^TM^ (Boston Scientific, Marlborough, MA, USA) device was implanted in the eight patients in this study (Figure 1). No significant postprocedural complications, as previously defined, were reported.

Short-term outcomes: During the first month, one patient experienced a clinically relevant non-major bleeding event (melena). In terms of antiplatelet therapy, it is important to emphasize that the best post-implant of LAAC management is still discussed, variable and adapted to each patient. After the procedure, three patients continued with OAT for two months and, subsequently, simple antiplatelet therapy with acetylsalicylic acid (ASA), with no bleeding events during the period that they were still under OAT. Three patients received DAPT, including ASA and clopidogrel for five weeks. Afterwards, two of them received ASA on a long-term basis, and the other one continued with DAPT because of percutaneous transluminal coronary angioplasty two weeks before LAAC. One patient received only clopidogrel because of an allergy to ASA. There was one non-procedure-related death four days after the procedure. Patient #7 presented good evolution during the first hours but, in the early morning, experienced cardiorespiratory arrest in asystole, with pulse recovery after 4 min of cardiopulmonary resuscitation (CPR) and two doses of adrenaline. Four days later, he died due to severe hypotension and multiorgan failure. Although no autopsy was performed, peripheral vascular complication, pericardial effusion and stroke were ruled out. 

**Figure 2:**
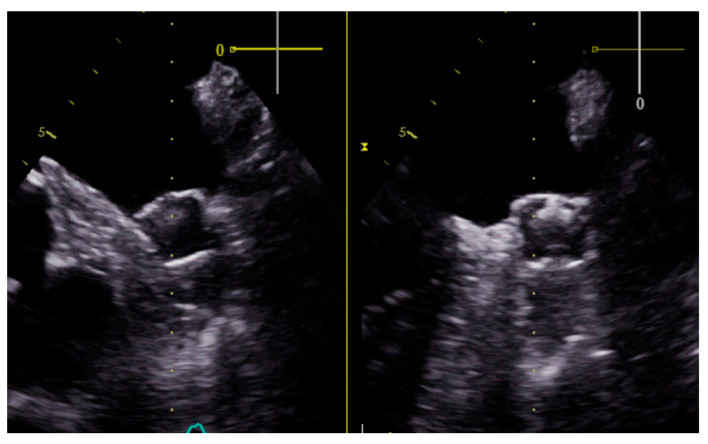
Transesophageal view of a Watchman FLX device in one of our patients three months after the left atrial appendage closure. The procedure was successfully completed, with complete exclusion of the appendage without complications or leaks exceeding 5 mm.

Long-term outcomes: During the subsequent follow-up period, with a mean duration of 14.7 months (SD ± 7.7), all patients were able to discontinue oral anticoagulation. No other deaths, new hemorrhagic or thromboembolic events were reported in any of the patients. A transesophageal echocardiogram (TEE) was performed in all patients 3 months after the intervention (Figure 2). One patient suffered a non-ST-segment elevation myocardial infarction six months after the procedure, which was managed conservatively, with good subsequent evolution. 

## 4. Discussion

This is the first study to evaluate the safety and effectiveness of LAAC with the Watchman FLX device as an alternative to OAT in a retrospective real-life 12-month follow-up cohort of eight consecutive patients with NVAF and CKD on MHD, who, due to their particular risk profile, were at high risk of thromboembolic and hemorrhagic events. 

Anticoagulation therapy is the standard of care approach for preventing thrombus formation and cardioembolic events in patients with NVAF. However, due to the increased risk of bleeding due to this therapy, some patients have absolute or relative contraindication for its use. Numerous studies underscore the lack of safety and efficacy of VKAs in patients with CKD, which is associated with an increased risk of bleeding, stroke and death [19,20,21]. 

VKAs could increase vascular calcification and aortic valve disease as an adverse side effect. This is due to the inhibition of the matrix Gla protein (MGP), which is a vitamin K-dependent protein acting as a local inhibitor of vascular calcification [22]. Currently, there is a lack of an antidote to reverse the anticoagulant effect for the majority of DOACs that could lead to a problem if the patient is a candidate for kidney transplantation. Thus, therapeutic alternatives must be considered. 

Since the advent of DOACs, comparative studies have consistently demonstrated their efficacy, on par with vitamin K antagonists (VKAs) but with a superior safety profile. Furthermore, they do not require continuous monitoring [23,24,25,26]. However, if we focus on patients with ESRD undergoing MHD, the evidence decreases, as very few studies have been specifically designed to address this population. The evidence is even more limited regarding the thromboembolic management of these patients with LAAC, a technique that has been demonstrated to reduce the incidence of thromboembolic events in the general population, with the added advantage of not increasing the occurrence of hemorrhagic events. 

The LAA, an embryological remnant, primarily regulates blood volume. It is located in close proximity to the left circumflex artery and has its upper boundary adjacent to the left superior pulmonary vein. The morphology of the LAA can vary significantly among patients, often presenting with multiple lobes. Under normal sinus rhythm, the LAA is a contractile structure that empties its content completely with each heartbeat. However, in cases of atrial fibrillation, the LAA loses its contractile function and begins to dilate. This dilatation results in slowed blood flow, leading to an increased risk of thrombus formation in this location [27,28]. Therefore, LAAC has been considered since the early 2000s as a potentially effective strategy for reducing the risk of cardioembolic events in NVAF patients.

In order to elucidate the impact of CKD on procedural success and clinical outcomes after LAAC, Della Rocca et al. [29] have recently published a study conducted at eight different international centers. They included 2124 AF patients undergoing LAAC and categorized according to the estimated glomerular filtration rate (eGFR) at the time of the implantation. LAAC effectively prevented thromboembolic and bleeding events, irrespective of the baseline kidney function, when compared with the expected rates for patients with similar CHA_2_DS_2_-VAS_C_ and HAS-BLED scores. However, with worsening baseline kidney function, a higher incidence of overall, but not major, peri-procedural complications was observed, and patients with moderate-to-severe CKD also had a higher incidence of the primary endpoint of CV mortality, stroke, transient ischemic attack (TIA), peripheral thromboembolism and major bleeding. 

Unfortunately, as mentioned before, scientific evidence is scarce regarding the use of LAAC in patients with ESRD undergoing MHD. There was a meta-analysis published in 2020 [30] that was conducted to assess the device’s efficacy in CKD non-HD patients with NVAF. It was observed that, despite the limited number of patients and the variability among the seven studies analyzed, this procedure could offer a safe and effective means of preventing the onset of stroke and TIA. It could potentially serve as a suitable alternative to pharmacological anticoagulation.

If we focus on patients with ESRD, the largest study is an Italian one, conducted at eleven centers, by Genovesi et al. [31]. They examined 92 patients with NVAF and ESRD on MHD who underwent LAAC compared to two patient cohorts, one on warfarin treatment (114 patients) and one without any treatment (148 patients). The devices were successfully implanted in all the patients, with no deaths or major adverse events at 30 days, and only three periprocedural minor bleeding events at the access site. Over the two years of follow-up, they found that in the LAAC cohort, the incidence of non-fatal cardiovascular events was significantly lower and the two-year survival rate was significantly higher. In terms of bleeding events, the incidence was significantly higher in patients on warfarin compared to the other two cohorts. The study suggests that LAAC is feasible and safe for dialysis patients and that in the long term, it is associated with better results compared to warfarin. These data coincide with the results of our cohort of patients.

Two recent studies have specifically evaluated this complex scenario. On the one hand, Fink et al. [32] collected patients from a German multicenter registry who underwent LAAC in order to evaluate the safety and efficacy of this technique in patients with ESRD. The study compared 57 patients with ESRD (defined as a glomerular filtration rate of <15 mL/min or chronic hemodialysis treatment) to a matched group of 57 patients without severe CKD. Frequency of major complications, defined as ischemic stroke/transient ischemic attack, systemic embolism, and/or major clinical bleeding, was 8.8% in patients with ESRD and 10.5% in matched controls (*p* = 0.75). This high complication rate is inconsistent with other studies, probably attributable to the inclusion of remote cases where the technique was not refined. The estimated event-free survival of the combined endpoint after 500 days was 90.7 ± 4.5% in patients with ESRD and 90.2 ± 5.5% in matched controls (*p* = 0.33). On the other hand, Ueno et al. [33] conducted a study in Japan in order to assess the feasibility of LAAC in HD patients with NVAF and high risk of thromboembolic stroke and bleeding. Peri-procedural and 45-day clinical outcomes after LAAC using the WATCHMAN system were retrospectively compared between 25 hemodialysis patients and 93 non-hemodialysis patients. All procedures were successful, except for a non-hemodialysis patient with a larger LAA. In terms of complications during the 45-day follow up, there was one hemodialysis patient with suspected bleeding and a non-hemodialysis patient who died due to cardiac amyloidosis. Therefore, LAAC appears to be both feasible and safe and effective in patients with ESRD and NVAF on hemodialysis.

Ongoing studies should provide further evidence to support the usefulness of LAAC in this group of patients. We look forward with optimism to the results of the EPIC06-WATCH-HD study, currently underway. It is an observational, prospective, multicenter study in which 51 patients were included. The primary efficacy endpoint is the combined criterion of embolic events (TIA, stroke and systemic embolism) and major bleeding events (Bleeding Academic Research Consortium > 2) at 24 months. The secondary safety objectives include major adverse events (mortality, stroke, systemic embolism, cardiac tamponade and pericardial effusion requiring intervention) and peri-intervention and device-related adverse events al 2 years (thrombosis, significant residual leak > 5 mm and embolization) (NCT03446794).

In our study, we obtained promising results, as the LAAC procedure was successfully performed in 100% of the patients. This efficacy in device implantation without periprocedural adverse events underscores the feasibility of this strategy in this high-risk group, confirming previous findings in similar patient populations [31,32,33]. The need to avoid bleeding risk in ESRD patients, as reflected by the high HAS-BLED score, was evidenced by LAAC being indicated in 75% of patients due to previous major bleeding episodes. This suggests that, in patients with a history of significant bleeding, LAAC can be an attractive option that eliminates the need for oral anticoagulation and further reduces the risk of bleeding events. In our series, all patients were able to discontinue OAT. Furthermore, our series includes patients at risk of bleeding as well as patients with an established contraindication to anticoagulation, making the therapy an attractive alternative in the management of these patients before they present a bleeding event.

This is the first series using the Watchman FLX implantation device exclusively and attests to its safety in this challenging population, as observed in global studies. It may aspire to achieve <1% of procedural complications [34]. 

Our series is a contemporary representative sample of the last decade with the exclusive use of the Watchman FLX device. In contrast, other studies with larger series, such as that based on a German registry [32] (13-year patient mix) and the study by Genovesi et al. [31] (inclusion period from 2010 to 2014, 4 years), included patients for a longer period of time and used more than one type of device.

The median dialysis time among the first four patients (59 months) versus the subsequent four patients (13 months) highlights the incremental confidence in outcomes and the role of the referring physician in achieving results. Intuitively, it would be important to consider sending patients for LAAC at the initial stages of their dialysis programs, anticipating situations of increased frailty. 

A crucial finding is that during long-term follow-up, no new thromboembolic or bleeding events were observed in any patient after discontinuation of oral anticoagulation. This supports the safety and efficacy of LAAC as a strategy for stroke prevention in this population, avoiding the potential risks of oral anticoagulation. It is important to emphasize that long-term follow-up in essential in this population, as their risk of thromboembolic and bleeding events persists over time. 

The viability of LAAC as an alternative to OAT in patients with NVAF and ESRD on MHD is of particular significance, as this population has been excluded from clinical trials supporting the use of OAT. Therefore, the importance of researching and presenting appropriate therapeutic options for this patient group, often in challenging clinical situations and lacking safe and effective alternatives, is emphasized.

However, it is crucial to recognize the limitations of this study, such as its small sample size, lack of a control group and reliance on retrospective data. Even so, our real-life experience serves to expand the available evidence and provides some genuine findings. Larger, controlled prospective clinical trials are needed to confirm these findings and provide a more comprehensive assessment of the efficacy and safety of LAAC as an alternative to oral anticoagulation in this high-risk clinical population.

## 5. Conclusions

LAAC as a non-pharmacological option for the management of NVAF in a small sample of patients with ESRD on MHD appears to be safe and effective for thromboembolic protection, with the advantage of not increasing the bleeding risk, which is already very high in this population. LAAC could represent a therapeutic alternative to OAT in dialysis patients with AF and high bleeding risk. The findings for this case series need to be confirmed in a larger prospective study with a longer follow-up period. 

## Figures and Tables

**Table 1:** Baseline characteristics before left atrial appendage closure.

	Patient 1	Patient 2	Patient 3	Patient 4	Patient 5	Patient 6	Patient 7	Patient 8
Age	86	83	81	85	78	80	74	64
Sex	Male	Female	Male	Male	Male	Male	Male	Male
Hypertension	No	Yes	Yes	Yes	Yes	Yes	Yes	Yes
Type 2 diabetes mellitus	No	No	No	No	No	Yes	No	Yes
Dyslipidemia	No	Yes	Yes	Yes	Yes	Yes	Yes	Yes
Smoker	No	No	Yes	No	Yes	Former	Yes	Yes
Time on dialysis until LAAC (months)	71	1	59	65	38	13	54	12
CHA_2_DS_2_-VASc	4	8	4	5	5	6	3	3
HAS-BLED	5	6	3	2	5	3	4	3
Previous stroke	No	Yes	No	No	No	No	No	No
Previous bleeding	Yes	Yes	No	Yes	No	Yes	Yes	Yes
	Hemorrhagic shock secondary to UGB	Hemodynamic instability secondary to GI bleeding	Labile INR	UGB	Anemia	GI and GU bleeding	Bleeding from dialysis vascular access	Hematuria, anemization
Permanent AF	No	Yes	No	No	Yes	No	No	No
Persistent/paroxysmal AF	Yes	No	Yes	Yes	No	Yes	Yes	Yes
Heart failure	No	No	Yes	No	Yes	Yes	No	No
Ischemic heart disease	Yes	No	Yes	Yes	Yes	No	No	No
Previous treatment								
- VKAs	Yes	Yes	Yes	No	No	No	No	No
- DOACs	No	No	No	No	No	Yes (Apixaban 2.5 mg/12 h)	Yes (Apixaban 2.5 mg/12 h)	Yes (Apixaban 2.5 mg/12 h)
- DAPT	No	No	No	Yes	Yes	No	No	No
Left ventricular ejection fraction (%)	50	60	30	33	51	59	60	60
Left atrium area (cm^2^)	50	45	48	38	39	21	32	30
LAAC device	Watchman FLX 27 mm	Watchman Flx 24 mm	Watchman Flx 24 mm	Watchman Flx 24 mm	Watchman FLX 24 mm	Watchman FLX 24 mm	Watchman FLX 31 mm	Watchman FLX 24 mm

LAAC: left atrial appendage closure. UGB: upper gastrointestinal bleeding. GI: gastrointestinal. GU: genitourinary. AF: atrial fibrillation. VKA: vitamin K antagonist. DOAC: direct oral anticoagulant. DAPT: double antiplatelet therapy.

**Table 2:** Results after LAAC.

	Patient 1	Patient 2	Patient 3	Patient 4	Patient 5	Patient 6	Patient 7	Patient 8
Intervention success	Yes	Yes	Yes	Yes	Yes	Yes	Yes	Yes
Follow-up (months)	17	26	12	14	26	12	….	10
Major or minor bleeding (<30 days)	No	No	No	Yes	No	No	….	No
Major or minor bleeding (>30 days)	No	No	No	No	No	No	…	No
Stroke (<30 days)	No	No	No	No	No	No	…	No
Stroke (>30 days)	No	No	No	No	No	No	…	No
Myocardial infarction	No	No	No	Yes	No	No	…	No
Thrombosis or leaks at 3-month control echocardiogram	No	No	No	Yes (leak < 3 mm)	No	No	…	No
Death	No	No	No	No	No	No	Yes	No
Post procedural treatment	ASA + clopidogrel 5 weeks	Clopidogrel	Acenocoumarol 2 months	ASA + clopidogrel	ASA + Clopidogrel	Apixaban 2.5 mg/12 h 2 months	ASA + clopidogrel	Apixaban 2.5 mg/12 h 2 months
Long-term therapy	ASA	Clopidogrel	ASA	ASA + clopidogrel	ASA	ASA		ASA

LAAC: left atrial appendage closure. ASA: acetylsalicylic acid.

## Data Availability

No new data were created or analyzed in this study. The data used to support the findings of this study are available from the corresponding author on request (contact M.E.B.V., mbasvel@mde.es or ebasabevelasco@gmail.com). I confirm that all the figures and tables are the original work of this manuscript’s authors. All figures and tables were produced by the authors of this manuscript; they were not adapted from other authors and do not include an online link.
